# Developing a Socioculturally Nuanced Systems Model of Childhood Obesity in Manhattan's Chinese American Community via Group Model Building

**DOI:** 10.1155/2020/4819143

**Published:** 2020-06-19

**Authors:** Ewelina Swierad, Terry T.-K. Huang, Ellis Ballard, Karen Flórez, Sheng Li

**Affiliations:** ^1^Columbia University Irving Medical Center, New York, NY, USA; ^2^Center for Systems and Community Design, Graduate School of Public Health and Health Policy, City University of New York, New York, NY, USA; ^3^Social System Design Lab, Brown School at Washington University in St. Louis, St. Louis, WA, USA

## Abstract

The purpose of this study was to develop a qualitative and socioculturally tailored systems model of childhood obesity in the Chinese American community in Manhattan's Chinatown. We utilized group model building (GMB) methodology as a form of participatory systems modeling. The study was conducted in Manhattan's Chinatown community. We recruited 16 Chinese American adults from the community. GMB workshops engendered a causal loop diagram (CLD), the visualization of a complex systems model illustrating the structures, feedbacks, and interdependencies among socioculturally specific pathways underlying childhood obesity, in Manhattan's Chinatown community. The analysis of CLD revealed that participants considered the following factors to influence childhood obesity: (1) traditional social norms affecting body image, how children are raised, parental pressure to study, and trust in health of traditional foods; (2) grandparents' responsibility for children; (3) limited time availability of parents at home; and (4) a significant amount of children's time spent indoors. GMB represents a novel method to understand the complexity of childhood obesity in culturally specific populations and contexts. The study identified sociocultural subsystems that may underlie the development and perpetuation of childhood obesity among Chinese American children. Insights from the study can be useful in the design of future empirical studies and interventions.

## 1. Introduction

In the last decade, increases in the prevalence of obesity have been documented among underrepresented ethnic groups [1] such as African American, Hispanic American, and Asian American children [2, 3]. Between 2002 and 2012, Asian Americans on the whole experienced 102% increase in obesity (5.1% to 10.3%) as compared to 35% increase among whites [4]. Although Asian American children have overall lower rates of overweight and obesity compared to other racial/ethnic groups in the US [3], they may be at higher risk for developing type 2 diabetes than other racial groups [5]. Importantly, national data regarding obesity rates rarely differentiate Asian American children of different cultural backgrounds [6, 7].

Indeed, based on limited data, significant subgroup differences in obesity rates among Asian American children exist. For example, Jain et al. [6] found that 34% of Vietnamese American children were either overweight or obese as compared to 26% of all Asian American children. Among Chinese American children, 23.5% of 4 year olds were found to be overweight or obese [6]. However, by the time they reach 6–11 years of age, nearly one-third was shown to be overweight or obese [2]. These trends suggest the “thin Asian” stereotype may be unwarranted [8] and call for the need to examine unique sociocultural pathways underlying obesity in different Asian American subgroups [9].

In light of the widening disparity in childhood obesity trends, there is a need to reevaluate a “one-size-fits-all” approach to obesity [10] and to consider the incorporation of sociocultural nuances in existing obesity prevention and treatment models. Culture influences obesity in myriad ways, including contributions to ideal child weight, child body image, child feeding practices, differences in levels and types of exposure to food marketing, preferences for and opportunities to engage in physical activity, and the utilization of health services, among other factors [9, 11]. In the context of Asian Americans, such models also need to take into account the varied experiences of migration and exposure to both the pre- and postimmigration environment [12]. Among Chinese Americans specifically, researchers and practitioners need to consider this group's unique immigration trajectories, prior experience with food insecurity that may contribute to parents' and grandparents' overfeeding practices [13], and the large amount of time that parents spend at work to support their families, which in turn affects children's food intake and physical activity patterns [14]. Understanding these socioculturally nuanced pathways to the development of obesity may be important to optimize the design and implementation of interventions [15].

Obesity is well understood to be complex, multilevel problems and previous studies have explored applications of systems modeling tools and frameworks to inform policy action [16–19]. However, many of these efforts have been expert-driven, relying on known literature and collaboration of a range of stakeholders, including scientists, the private sector, and government departments, as opposed to community members. Yet in order to explore the complex, locally driven, and culturally specific dynamics of interest to this study, a generalized and expert-driven causal map is insufficient to meet our needs.

Group model building (GMB) offers a bottom-up, participatory strategy representing a method that engages diverse voices in communities to cobuild the complex systems maps of obesity causes, to codesign potential solutions, and to inform socioculturally nuanced theoretical models of childhood obesity [20, 21]. GMB is one type of participatory systems modeling method that seeks to engage stakeholders in the process of informing model construction [22]. From its foundations, system dynamics has emphasized the importance of multiple sources of data for informing model structure, including quantitative data, qualitative and documentary records, and what Forrester describes as the “mental database” of experiences and observations of those working and living in systems [23]. Approaches for engaging stakeholders in model construction range from key informant interviews and focus groups to multisession scripted participatory group model building workshops [24]. The specific tradition that informs this study is Community-Based System Dynamics, in which participants themselves “hold the pen” and draw model structure from their own experience and the goals of group model building are both to inform model structure and to build capabilities within communities to understand the tools of system dynamics and systems thinking [20]. In all of these traditions, the specific exercises and diagramming tools, including causal loop diagrams, stock and flow diagrams, and behavior-over-time graphs, are used as methods to elicit stakeholder perspectives to craft a hypothesis of system structure.

Through a collaborative effort, a structured set of modeling exercises can be used to codevelop a causal loop diagram (CLD) to reveal interconnections between tangible and intangible variables. The CLD articulates key (locally specific) structures and feedback loops in the obesity system and often leads to new hypotheses, which are useful for further empirical research. GMB has demonstrated its usefulness in exploring sociocultural factors that affect health, including healthy eating, active living, and childhood obesity [25–29]. In addition to other GMB strategies implemented and described below, CLD was adopted as a method that is aptly applicable for exploring complex problems such as obesity and for uncovering visual and dynamic interconnections between factors contributing to childhood obesity. CLD is a standard technique in systems dynamics modeling, upon which GMB is based [20]. This methodology has a practical application enabling public health researchers, practitioners, and community stakeholders to collectively visualize the connections between multiple dynamic variables and to design health promotion solutions that addresses obesity from the community perspective. This approach is useful for developing successful community-level interventions [30].

In this study, we sought to develop a socioculturally nuanced model of childhood obesity in the Chinese American community in Manhattan's Chinatown in New York City (NYC). The purpose of this paper is to describe the GMB method used and to present the main findings based on the insights gained from the CLD.

## 2. Materials and Methods

### 2.1. Population and Settings

Study participants were selected from local residents with help from the largest NGO and the largest nonprofit healthcare provider network in Manhattan Chinatown. Sixteen Chinese American adults volunteered to attend the GMB workshops and each of them was offered $50 for participating. All participants were aged between 20 and 60 years, and 43.8% (7/16) were male. Six participants were born overseas. Ten individuals attended both sessions while six individuals attended one session. The participants represented a variety of occupations including nurses, school guidance counsellors, restaurant owners, community health workers, and housewives. Asian Americans represent the fastest growing racial group and Chinese Americans are the largest subgroup in the USA and in NYC between 2000 and 2010 [31]. The growth of Chinese Americans is largely by immigration and Chinese immigrants are less likely to be proficient in English [31]. In NYC, more than half of Chinatown residents are foreign-born.

In February 2017, our research team worked with the Charles Wang Community Health Center in Chinatown, Manhattan, New York City, to organize two half-day GMB workshops. One of the team members communicated with the local community and set up the materials. GMB flyers were prepared in both English and Chinese and distributed to solicit potential research participants.

This study was considered exempt by the CUNY Graduate School of Public Health and Health Policy Institutional Review Board.

### 2.2. Study Design and Analysis

The research team consisted of five individuals with expertise in participatory system dynamics modeling, obesity, epidemiology, community health, and the Chinese American culture. Prior to the GMB workshops, the research team convened a series of 4 conference calls to design the protocol of the workshop and to develop a facilitation manual that described in detail each structured activity or “script” selected for the workshop along with the allotted time and facilitation processes [32–34]. The research team ensured that each activity proposed for the workshop was culturally appropriate to the stakeholders and promoted “a facilitative attitude” [35]. A detailed agenda was also developed to accompany the facilitation manual (see https://bit.ly/2Dx0jPe).

The workshops started with an introduction by the research team, followed by a brief presentation about the problem of childhood obesity in Chinese American children and an overview of the workshop goals. To alleviate the barriers to participation (e.g., language, literacy, and social status), the workshop was led by individuals connected to the communities in Chinatown, rather than a group of complete outsiders. English was used as the main language, though Chinese translations were used at times. Throughout the duration of the workshop, the participants engaged in a series of exercises—outlined by the scripts developed for the workshop—to identify interconnected factors contributing to childhood obesity among Chinese Americans (see Table 1 for a summary of key activities).

The first day of the workshops concluded with a preliminary CLD, where the participants were able to review and discuss CLD. The research team then worked overnight to summarize and clarify the CLD using a system dynamic modeling software Vensim PLE (Ventana Systems, Harvard, MA). The next step was to highlight balancing and reinforcing feedback loops, linking them to the key stories identified during the workshop.

On the second day of the workshop, the research team presented the synthesis of the CLD for review by the participants. The participants then examined the structure and contextual narratives of the CLD, adding and editing loops. They then deliberated on any misrepresentations or misunderstandings portrayed in the model. As participants reviewed the model, a modeler revised the causal structure in real time on a projected version of the CLD. The following step involved the activity focused on eliciting intervention or action ideas that target the system structure to reduce or prevent rises in childhood obesity. These ideas were mapped onto the revised model in real time, offering participants opportunities to address multiple intended and potentially unintended consequences of these interventions. The team then closed the session by thanking attendees for their participation and describing how the ideas and model generated during the workshop would be used to inform future research.

## 3. Results

**Figure 1:**
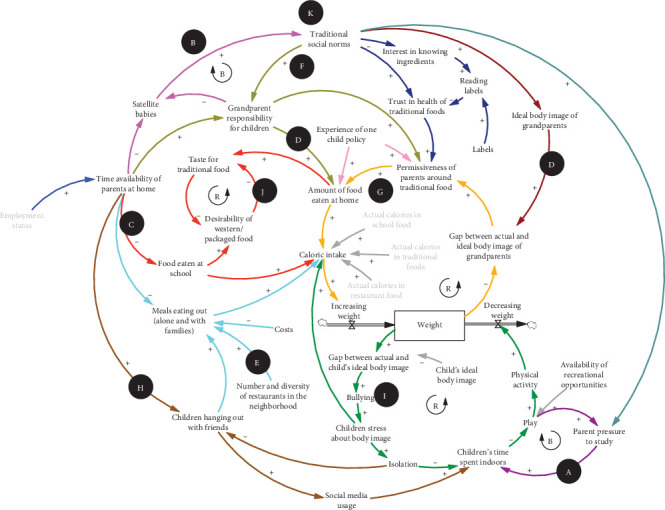
Causal loop diagram (CLD) of childhood obesity in the sociocultural context of the community in Manhattan's Chinatown. 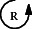
and 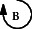 refer to reinforcing and balancing feedback loop, respectively. This CLD presents the local residents' view of childhood obesity-related factors and links among them. A through K illustrate subsystems of factors, links, and feedback loops.

A synthesis of the CLD is presented in Figure 1. This figure illustrates subsystems, factors, and feedback loops that influence childhood obesity, specifically food intake and physical activity practices among Chinese American children in Chinatown neighborhood in NYC. As presented in the figure, the CLD provides a visual depiction of the components of the system and their interconnections, highlighting the patterns of obesogenic behavior within the unique sociocultural context of Manhattan's Chinatown community. CLDs highlight the endogenous perspective, a hallmark of system dynamics that seeks to understand system behavior, including patterns of health and wellbeing within a community, as a product of system structure, or the specific interconnections of components and parts that comprise the system. These interconnections take the form of balancing (B) loops (indicated by odd number of negative signs), in which system structure opposes or resists change, tending toward stabilization or goal-seeking behavior, and reinforcing (R) loops (indicated by zero or even number of negative signs), in which changes in a variable are amplified, resulting in exponential growth or decay. From this view, a system's response to external perturbations such as policy changes, interventions, or macroeconomic shifts is a product of the specific combination of feedback loops described in a CLD. The relative strength of balancing and reinforcing loops changes as a result of accumulations of variables within the system. The CLD therefore represents a hypothesis of the system structure in which causal chains can be traced, but a qualitative diagram alone does not make statements about the relative strength or weight of any one variable, causal link, or loop at any point in time.

### 3.1. Major Factors and Structures

Key themes emerging from the CLD that were perceived to influence childhood obesity were as follows: (1) traditional social norms affecting what is considered typical body image, how children are raised, parental pressure to study, and trust in the health of traditional foods, (2) grandparents' responsibility for children, (3) limited time availability of parents at home, and (4) a large amount of children's time spent indoors. The key balancing feedback loops and reinforcing feedback loops are further described as follows.

#### 3.1.1. Cultural Preference for Sedentary Behavior, Strong Academic Pressure versus Physical, and/or Outdoor Activity

Participants identified strong academic pressure as one of the most important factors contributing to sedentary behaviors among Chinese American children. As participants reported, given that the Chinese American culture highlights the importance of education and academic performance, children spend a considerable amount of time indoors studying, and as a result, they have a little time left for physical activity. The more time they spend studying, the less time they have for playing and other activities, which, in turn, makes them feel more isolated from their peers and compromises the amount of time they spend with their friends. Consequently, they turn to social media to stay connected with others. This frequent online activity further reinforces children's physical inactivity and contributes to weight gain among Chinese American children.

#### 3.1.2. Children Separated from Parents (Due to Parents' Distant Employment and the Satellite-Baby Phenomenon)

As parents' ability to take care of their children decreases due to employment status and work responsibilities in the US, the role of grandparents in raising children increases. As a result, grandparents assume great responsibilities for taking care of young children and shaping their health-related practices such as food intake and physical activity. The “satellite baby” phenomenon exemplifies this shift of responsibilities, particularly among newly immigrant Chinese families. This phenomenon involves Chinese American children born in the US, but moving back to China to stay with their grandparents. Parents' demanding work hours and high financial costs of childcare in the US are two main reasons for the move. Another reason involves the parents' desire to instil Chinese traditional cultural values in their children. The satellite baby phenomenon could have implications for children's health. Because of the cultural value placed on expressing care and love through food, grandparents tend to overfeed their grandchildren, which may consequently lead to weight gain among Chinese American children [36].

#### 3.1.3. Children Separated from Parents (Food Eaten at Home versus at School)

Consistent with prior literature, we have found that Chinese immigrant families are often dual earners. Consequently, they are very busy with their jobs and are sometimes challenged with a distant employment reality. Many new immigrants tend to leave their families in NYC as a result of working in other states. Because of the distant employment phenomenon and consequently limited time for their families, parents rarely engage in preparing healthy foods for or exercising with their children. As a result, children often consume meals at after-school programs, and the quality of these meals is variable as their nutritional value is not regulated the same way as USDA-subsidized breakfasts and lunches. Consumption of often-unhealthy food during after-school programs increases children's desire to consume highly processed, western packaged food and decreases their preference for some of the healthier traditional food. Similarly, because of parents' limited time at home, children eat out more frequently, particularly when it is a way for them to spend time with friends in Chinatown. These practices may lead to excessive caloric intake and contribute to children's being overweight.

#### 3.1.4. Pressure to Feed from Grandparents (Due to One-Child Policy's Impact or Beliefs about Chubby Babies as Healthy)

Participants discussed how the one-child policy and cultural beliefs surrounding ideal body image for children affect grandparents' overfeeding practices and perception of what it means to be healthy, and what is the ideal and culturally valued body weight for their grandchildren. Equating chubby babies to healthy babies was identified as one of the major problems that contribute to grandparents overfeeding their grandchildren. These overfeeding practices are fueled by grandparents' belief that the heavier a child, the more love and care are involved in raising that child [37]. In addition, the one-child policy in China led to concentration of grandparents' attention on the only grandchild in the family, increasing overfeeding tendencies further.

#### 3.1.5. High Diversity and Number of Food Outlets in Chinatown

In Manhattan's Chinatown, the density and diversity of restaurants, food retail shops, and grocery stores are higher than in common neighborhoods in the US. Participants considered the exposure to easily accessible and diverse food outlets in Chinatown as one of the factors that influences increased consumption of a large amount of food in their families. Frequent consumption of high caloric food easily available in the neighborhood reinforces children's weight gain. Unhealthy eating practices triggered by the abundance of nutritionally compromised foods are further reinforced by the lack of proper information about the quality, portion size, and nutritional value of the foods that Chinese Americans consume and feed their children with.

#### 3.1.6. Beliefs about Healthfulness of Traditional versus Western Foods (Especially Snacks)

Participants reported that people in the community do not often analyze the nutritional value of their traditional meals to distinguish between healthy and unhealthy food. This practice has consequences for their and their children's health because some of the traditional dishes may be highly caloric or prepared through unhealthy cooking methods. The general perception is that all traditional foods, even snacks, are better for health than Western foods.

#### 3.1.7. Parents' Experience of Food Scarcity

Participants discussed how preimmigration experiences of food scarcity coupled with postimmigration exposure to an abundant food environment have influenced the way in which Chinese Americans feed their children. Prior experience with food insecurity may contribute to parents overfeeding their children, especially in a food-dense, obesogenic environment [13].

#### 3.1.8. Traditional versus Western Ideals of Independence among Youth (Peer-Influence)

Participants reported that Chinese American children experience cultural clash between traditional versus Western ideals of independence. This cultural conflict expresses itself in the relationship between Chinese American children and their American peers as well as between children and their parents. Given that Chinese American children aspire to feel more independent just as their American counterparts [38], they experience conflict with their parents who want them to be more traditional and less autonomous. This value discrepancy influences children's health behaviors and psychological wellbeing. The issue is complicated further due to parents' busy work schedules that result in children spending a lot of time alone in front of their electronic devices and that, in turn, creates many opportunities for them to snack and overeat.

#### 3.1.9. Bicultural Conflict (Asian versus Western)

Participants reflected on Chinese American children's experiences of bicultural conflict and its health consequences, particularly concerning norms and values surrounding body image. As the participants discussed, Chinese American children feel confused about what constitutes beauty and what is desirable body shape because the images that they are exposed to promote predominantly Western ideals of “healthy” and “beautiful” body types that are often unattainable and culturally irrelevant for Asian American children. As a result, Chinese American children are left with a lack of culturally sound role models who would promote healthy body image that is congruent with children's Asian American identities and their body types. Experiencing such cultural invalidation with regard to their body image, Chinese American children can struggle with self-esteem that further trigger unhealthy behaviors and compromise psychological wellbeing. Distress related to navigating the environment that is not culturally nuanced and supportive of children's values coupled with exposure to the mainstream ideals of beauty and health [36] may influence children's food intake (e.g., overeating as a form of coping, dieting) and physical activity practices.

#### 3.1.10. Dislike of School Lunch and High Consumption of Poor After-School Foods

Because of the fact that lunches offered at schools may not appeal to children's taste and preferences for traditional cuisine, Chinese American children engage in frequent consumption of highly processed, packaged, and unhealthy Western snacks. Participants perceived school lunches as lacking Asian flavors that would encourage their children to consume balanced and healthy meals. In the same vein, as mentioned above, Chinese American children often consume unhealthy meals during after-school programs, which further contributes to their weight gain [38].

#### 3.1.11. Strong Affinity for Local Primary Care

Participants expressed their trust towards local primary care (in the Chinatown community) and the need to implement health education in the community healthcare setting. This education should be delivered in a culturally sensitive, bilingual manner, incorporating individuals' values, and beliefs about health and culturally preferred health behaviors. Such culturally sound services can in turn decrease the risk of childhood obesity.

### 3.2. Structures Insights from Multiple Feedback Loops

The narratives above can be interpreted through the lens of feedback structures that provide insight into participants' perspectives on the boundaries of factors that can be influenced or changed, as well as some of the dominant feedback structures that either stifle or perpetuate change. The combination of different themes identified by the participants form feedback structures (i.e., reinforcing and balancing loops) that contain multiple narratives (discussed above).

The GMB workshop revealed the pressure toward high caloric consumption in terms of volume of food consumed (both within the house and outside) and a potential erosion of the nutritional value of foods in both settings. There is pressure among Chinese American children toward consuming food more frequently outside (restaurant and snack food), and there is a tendency in this community to consume larger quantity of food at home. The pathways through which weight gain occurs suggest a compounding influence of less nutritious food through snacks and fast food, as well as a continued push toward overfeeding.

The results of the workshop also suggest the lack of or weak balancing loops to control unhealthy behavior. This outcome is exemplified by strong reinforcing loop of taste of western food, which means that school lunch—nominally intended to be a nutritional supplement—may undermine other food interventions. Similarly, parental pressures toward studying may undermine children's healthy action to exercise, their fitness, or just time outside.

The balancing mechanism of grandparents may resist efforts by parents or children to maintain more healthy weight. Action to adjust children's weight is oriented around culturally circumscribed goals of ideal body weight. Traditional norms, parents' time with children, and nutrition of food available are considered to be factors that are exogenously driven, or not influenced by feedback dynamics within the model. All those identified patterns can affect food decisions and risks, perpetuating unhealthy food patterns and strengthening reinforcing loops. However, the assumptions of the exogeneity of these loops should be questioned and perhaps challenged through additional work on the feasibility of norm changes or potential interventions to link systems behavior to economic or social conditions at the community level.

### 3.3. Implications and Tailored Intervention Levers Informed by CLD

Insights from the CLD can inform culturally sensitive health promotion strategies for Chinese American families that otherwise might have been difficult to conjure. As presented in Table 2, to tackle the sedentary behaviors among Chinese American children, health promotion endeavors could capitalize on the cultural value placed on education within this ethnic group. Specifically, because athletics are important for college application, health promotion campaigns could frame good nutrition and active lifestyle (e.g., joining sports) as an academic goal to increase the chances of getting into a college. To mitigate some of the negative influences of grandparents on children's food intake, grandparents in both the US and China need to be provided with appropriate, culturally sound nutrition education, which can be delivered through locally based social networks and media. Pertaining to obesogenic aspects of the Chinatown environment, local restaurants and retailers may need to be involved in efforts against obesity. For example, local food businesses could make the calorie and nutrition content of Asian foods transparent, and health entrepreneurs can create healthy alternatives to what has been already available in the area. To address the issue of inadequate health awareness and false beliefs about healthfulness of traditional versus Western foods, the calories, and nutritional content of traditional, unpackaged foods may need to be made more transparent, in conjunction with food labeling education in both English and Chinese for Chinese American families.

Education about the experience of immigration and children's exposure to the obesogenic environment was identified as potentially helpful ways to address food scarcity experiences among Chinese American parents and grandparents. To mitigate the influence of the cultural clash on Chinese American children's health, health practitioners could implement the strategies involving youth cultural advocacy, strong leadership, and cultural voice to promote health, wellbeing, and independence among Chinese American children. Regarding bicultural conflict, health promotion efforts could focus on culturally sensitive health campaigns that incorporate Asian identity-enhancing strategies and Chinese American role models who would promote and illuminate culturally valued health behaviors and body image. Finally, to improve after-school menus, Asian flavors could be introduced to align with culturally specific taste preferences.

## 4. Discussion

To our knowledge, this was the first study using GMB as a method to develop a culturally tailored theoretical model of childhood obesity in a Chinese American community. The unique pathways underlying childhood obesity in Manhattan's Chinatown represent new hypotheses that can be examined further through additional empirical research. GMB was also useful in bringing diverse stakeholders together to develop a system understanding of the problem, thus paving the way for further collaboration and community action. Our research contributes to the literature on immigrant and minority health. We acknowledge that GMB is only the first step to inform future modeling efforts and intervention design and planning. However, GMB is an increasingly recognized and used technique in public health to advance both the researchers' and the community's shared understanding of the complexity of a problem [26]. The GMB process provided us with an opportunity to explore this issue in a unique, interactive, and culturally sensitive manner allowing us to obtain narratives that deeply resonate with the community of interest. More importantly, the process was helpful to involve community stakeholders to develop a shared understanding of the problem at hand. The CLD output from GMB is useful in identifying the complex structures and feedback loops underlying the phenomenon; these are known to be difficult to elicit using traditional public health methods such as surveys and standard focus groups [39]. Outputs from GMB can be considered as providing a novel set of testable hypotheses which can inform further empirical and intervention work.

The CLD generated through our study suggested nuanced findings that have not yet been explored previously in the literature. These insights include (1) hypotheses from the model that could be tested through further empirical research (e.g., impact of food eaten at school influencing norms and acceptability of western/packaged food, elasticity of grandparents' food norms, diversity of grandparents' ideal body image for children, or beliefs in health of traditional foods); (2) explorations of cultural practices that have been understudied (e.g., grandparents' dietary practices – and the role of nutrition education – in the US and China); and (3) culturally tailored implementation science research of known interventions (e.g., after-school interventions focusing on sports in addition to scholastics, Chinese-language food labeling interventions, peer-influence interventions focused on parents and grandparents, etc.). As discussed in the implications section above, the results of the current GMB study highlighted a range of interconnected factors that influence the development of obesity among Chinese American children. The insights from the systems map can inform more nuanced understandings of the pathways through which culturally sensitive health promotion strategies—designed for Chinese American children—may operate, as well as enabling or inhibiting conditions that may explain success or failure of future interventions. The current findings illustrate how it is critical to move beyond standard health communication approaches and guidance that may not be relevant or specifically tailored to this community context and implement culturally sound strategies that reflect the voice of the community and its specific concerns.

Researchers and health practitioners need to recognize health disparities among different groups of Asian Americans that are often overlooked partly because of the “thin Asian” [12] and “model minority” stereotypes that mask cultural and health variations among different Asian American subgroups [12, 40]. Although obesity rates are lower in Asian American groups as compared to other ethnic groups [3], Asian Americans suffer disproportionately from obesity-related diseases such as type 2 diabetes [41]. In addition, Asian American children born in the US have greater obesity risk than foreign-born youth, with obesity rates similar to nonimmigrant youth [42], further exacerbating risk for diabetes and cardiovascular disease.

Childhood obesity among Chinese American children is on the rise [4]. Some research suggests that one-third of 6-to-11-year-old Chinese American children are overweight or obese [9]. Moreover, although traditional Asian diets rich in fruit and vegetables may protect against many chronic diseases, qualitative research has shown that Asian American children prefer and regularly consume many of the unhealthy Western foods (e.g., sweetened beverages, pizza) in lieu of healthier traditional food options [14]. Research has also shown that Chinese American adults are at higher risk for hypertension than white Americans [43]. Less than half of elderly Chinese Americans aged 66–92 years with hypertension have their blood pressure under control [44]. Because Chinese Americans represent the fastest growing minority group and the largest Asian American subgroup in the US, more health research specific to this population is warranted.

It is critical to recognize heterogeneity within different Asian American subgroups and to explore unique sociocultural factors associated with obesity in different groups [9]. Pathways to health disparities can vary significantly among subgroups of Asian Americans, including differences in socioeconomic status, access to resources, migration patterns, immigration histories, and cultural norms [45]. For decades, health services for Asian American populations have been hampered by the myth that all Asian Americans have lower burdens of disease than other ethnic groups [46]. This myth was, in part, perpetuated by the lack of reliable data and the lumping of Asian Americans into one large cluster when in fact they represent a heterogeneous group [6, 47]. Just as there is a great within-group variability among African Americans [48] and Hispanic Americans [49], not all Asian Americans are the same [4, 47, 50].

To date, most current strategies that address childhood obesity have not been effective [51] which can be partly attributed to the lack of culturally specific models of obesity [14]. Improved culturally based models can help to identify unique levers and to be key to intervention design and effective implementation [14]. Recent research suggests that culturally adapted obesity prevention interventions may be more effective in addressing childhood obesity than generic campaigns [52]. In the context of Chinese American children, consistently with the results of our study, Diep et al. [14] argued that it is critical to design interventions that are culturally relevant and that target parents, grandparents, and extended family to encourage modeling of healthy eating and other effective food practices. In addition, future research needs to identify the best ways to account for acculturation to balance healthy aspects of Asian and non-Asian diets, recognizing that cultural assimilation is an inevitable part of the immigrant experience [14].

Our workshop has demonstrated that GMB that allows for the identification of the rich structure and deep meanings associated with a particular problem [53] is a useful and culturally sensitive tool for unpacking the complexity of obesity and associated health disparities. GMB provides a way to conceptualize childhood obesity in a participatory manner and to offer insights about health interventions. During our workshop, we observed that by ascertaining the feedback loops, it became immediately clear to community members that childhood obesity was a multifactorial problem and that many interdependencies existed to give rise to the issue. Past research has shown that such elucidation is key to decision-making and prevents key stakeholders from resorting to simplistic, silver-bullet strategies that rarely work [9, 54]. In our workshop, we used GMB solely as a qualitative tool to navigate, challenge, and enhance our mental model as a starting point to address obesity among Chinese American children. In the future, results of the model can be supplemented by additional empirical work such as surveys to calibrate the system parameters and feedback loops. The model can be subjected to computational simulations and used as a virtual laboratory for intervention and policy design and testing.

Although the results of the current study are informative, some limitations need to be acknowledged. This is a pilot study and the results need to be further validated by follow-up research (e.g., surveys, in-depth interviews) exploring the health behaviors of Chinese American families in detail. Because the study was conducted in Manhattan's Chinatown, the results may uniquely represent the perceptions, values, and behaviors of Chinese Americans living in this particular area. Findings may not be generalizable to other Chinese American communities or to other Asian American subgroups. Future studies to replicate the approach in other Asian American subgroups are warranted.

## 5. Conclusions

Asian Americans are heterogeneous and experience obesity-related health disparities, and more socioculturally models of obesity are needed in specific Asian subgroups. This paper presents GMB as a novel method to tackle the complexity of major public health challenges such as childhood obesity in culturally specific populations and contexts. In particular, the study led to a new understanding of the sociocultural subsystems that may underlie the development and perpetuation of childhood obesity in the Chinese American community in one part of New York City. Insights from the study are useful for the design of future interventions in this specific community. Socioculturally tailored interventions may be more effective and sustainable in reducing health disparities.

## Figures and Tables

**Table 1:** The script choice, purpose, and outcomes.

Script	Purpose	Outcomes
Hopes and fears	To elicit and to establish group expectations for a GMB session	List of participants' hopes and fears with regard to GMB workshop

Graphs over time	To engage participants at the beginning of GMB session in framing the problem, initiating mapping, eliciting variables, and gathering input in deciding the reference modes for the workshop	Candidate variables for the dynamic model or the map

Dots	To vote for what participants considered to be the most important drivers of the modeled problem (i.e., childhood obesity, trends over time identified in the graphs over time exercise)	Prioritized choices and the rank of importance of factors contributing to the problem development and persistence over time (i.e., drivers of childhood obesity)

Causal-loop diagram (CLD)	To explore the causal factors and the consequences of childhood obesity among Chinese Americans in their community; to pinpoint connections and the nature of the relationship between these factors; and to visualize various interactive causal pathways affecting the weight status of Chinese American children	A causal loop diagram which may be described either in a report (in the case that only a qualitative model is built), or be used as a dynamic hypothesis on the basis of which formal modeling starts

Model review	To present a preliminary synthesis causal loop diagram for review and critique by workshop participants	A revised causal loop diagram and articulation of key feedback loops, narratives, themes, and group understanding of the correspondence between model structure and system behavior

Action ideas	To generate a list of intervention ideas based on the causal structure from the synthesis causal loop diagram	A list of intervention ideas including a description of how those intervention ideas may act on the system, including feasibility and barriers and facilitators to impact

**Table 2:** Unique sociocultural constructs and tailored intervention levers.

Unique sociocultural constructs	Tailored intervention levers
(A) Cultural preference for sedentary behavior, strong academic pressure versus physical or outdoor activity	Frame physical activity skills as necessary for academic Achievement; social campaign to frame academic success as a function of good nutrition and active lifestyle; more community and team-based outlets for physical activity and sports

(B) Children separated from parents (due to distant employment and satellite-baby phenomenon)	Grandparent nutrition education both in the US and China

(C) Children separated from parents (food eaten at home versus at school)	Grandparent and parent nutrition education both in US and China

(D) Pressure to feed from grandparents (due to one-child policy impact or beliefs about chubby babies as healthy)	Grandparent and parent education about health and nutrition; target grandparents through locally based social circles and media to alter climate of attitude

(E) High diversity and number of food outlets in Chinatown	Involvement of local restaurants and retailers in social campaign on portion sizing; make transparent the calorie and nutrition content of Asian foods; create competitive playing field for local food outlets to be recognized as health entrepreneurs

(F) Beliefs about healthfulness of traditional vs. Western foods (especially snacks)	Making transparent the calorie and nutrition content of traditional, unpackaged foods; educational campaign for schoolchildren and adults on quality of traditional foods and nutrition label reading

(G) Parents' own experience of food scarcity	Parenting education about the experience of immigration and children's exposure to the obesogenic environment

(H) Traditional versus Western ideals of independence among youth (peer-influence)	Advocacy intervention through youth leaders

(I) Bicultural conflict (Asian versus Western)	Build esteem around Asian identity; role modeling

(J) Dislike of school lunch and high consumption of poor after-school foods	Introduce Asian flavors to school lunches; improve after-school menus

(K) Strong affinity for local primary care	Obesity education for local primary care; deliver educational messaging to patients through primary care

## Data Availability

All requests for data access should be addressed to the corresponding author. Proposals requesting data access will have to specify how they plan to use the data.
